# Draft genome sequence data of multi-stress tolerant yeast *Millerozyma farinosa* KCTC27753 isolated from nuruk

**DOI:** 10.1016/j.dib.2022.108030

**Published:** 2022-03-08

**Authors:** Jeong-Ah Yoon, Yu-Jeong Lee, Eun-Hee Park, Hun-Joo Kwon, Myoung-Dong Kim

**Affiliations:** a Department of Food Biotechnology and Environmental Science, Kangwon National University, Chuncheon 24341, Korea; b Department of Food Science and Biotechnology, Kangwon National University, Chuncheon 24341, Korea; c Institute of Fermentation and Brewing, Kangwon National University, Chuncheon 24341, Korea

**Keywords:** Genome sequence, *Millerozyma farinosa*, Stress tolerance, Fermentation inhibitor, Nuruk

## Abstract

The strain *Millerozyma farinosa* KCTC27753, isolated from nuruk, is a multi-stress tolerant yeast which grows at 46 °C temperature and pH 3.0. This strain can withstand fermentation inhibitors, such as furfural and phenolic compounds released from biomass. Hence, this strain could be used for bioethanol production. The draft genome sequence of *M. farinosa* KCTC27753 was analyzed by PacBio RSII. The genome length is 21,255,474 bp and it consists of 17 contigs. The GC content of the genome is 41.1%. The genome analysis identified a total of 10,910 plausible gene-coding regions in this strain.

## Specifications Table

**Table:** 

Subject	Genetics: General
Specific subject area	Genomics and Molecular Biology
Type of data	Table and Figure
How the data were acquired	The draft genome sequence was determined using a Pac-Bio RSII instrument. The reads were assembled *de novo* into 17 contigs using FALCON (v0.2.1). The open reading frames were predicted using MAKER (v2.31.8).
Data format	Raw and analyzed
Description of data collection	*M. farinosa* KCTC27753 was isolated from nuruk produced in Kangwon-do, Republic of Korea. The genomic DNA was extracted from a pure culture of the strain and then used for sequencing.
Data source location	• Institution: Kangwon National University • City/Region: Chuncheon, Kangwon-do • Country: Republic of Korea • Latitude and longitude: 37°87′ N and 127°74′ E
Data accessibility	The draft genome sequence of *M. farinosa* KCTC27753 is available at DDBJ/EMBL/GenBank under the BioSample, BioProject, and assembly/WGS accession numbers SAME06289667, PRJNA369593 (http://www.ncbi.nlm.nih.gov/bioproject/PRJNA369593), and GCA_002196765 (https://www.ncbi.nlm.nih.gov/assembly/GCA_002196765.1), respectively.
Related research article	• H.J. Kwon, M.D. Kim, Isolation of stress-tolerant *Pichia farinosa* from nuruk, *Microbiol. Biotechnol. Lett*. 44(2016) 349-354.

## Value of the Data

• The draft genome of *M. farinosa* KCTC27753, isolated from nuruk, could reveal intricate genetic information of the strain. This would promote the understanding of the various genetic attributes of the strain.
• The draft genome data of *M. farinosa* KCTC27753 could promote understanding of multi-stress tolerant yeast.
• The genome sequence data would be useful in identifying and characterizing *M. farinosa* KCTC27753 strain-specific enzymes and gene clusters.

## 1 Data Description

The necessity of recoverable bioethanol production using microorganisms is globally on the rise because of the rapid exhaustion of fossil fuel and crude oil, hike in fuel prices, and environmental problems [2,7]. The multi-stress tolerant yeasts play pivotal role in fermentation-mediated ethanol production [5]. The *M. farinosa* KCTC27753 strain grows at temperatures as high as 46°C and can withstand a pH as low as 3.0. The multi-stress tolerant properties of this strain makes it suitable for ethanol fermentation as it can reduce the cooling cost for saccharification [10]. Moreover, this strain can also withstand fermentation inhibitors, such as, furfural and phenolic compounds released from biomass [3,6]. All these properties make this strain an ideal candidate for bioethanol production.

**Fig. 1:**
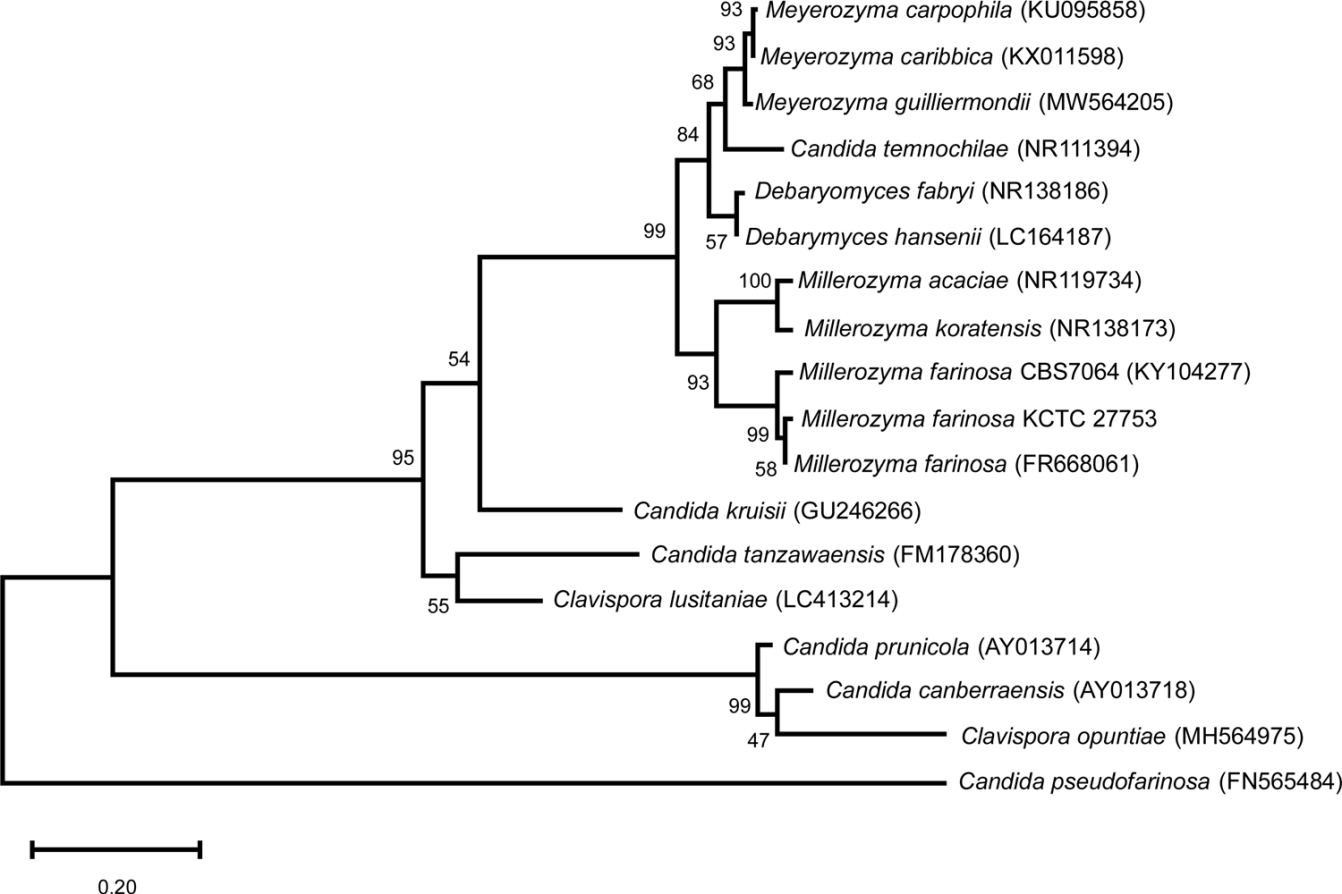
Phylogenetic tree of the *M. farinosa* KCTC27753 ITS region in correlation with other yeasts. The tree was constructed using the neighbor-joining method [9]. The numbers on the nodes correspond to the percentages, with which clusters appeared in bootstrap tests based on 1000 pseudoreplicates. The bars denote the relative branch lengths. The ITS regions were identified by GenBank accession numbers (shown in parentheses).

Fig. 1 shows a phylogenetic tree of the internal transcribed spacer (ITS) region of *M. farinosa* KCTC27753 and other yeasts. The ITS region of *M. farinosa* KCTC27753 showed the highest similarity to that of *M. farinosa* CBS8045.

Table 1 summarizes the features of the draft genome sequence of *M. farinosa* KCTC27753 and *M. farinosa* CBS7064 strains [8]. The *M. farinosa* KCTC27753 genome contained 21,255,474 bp distributed across 17 contigs with a GC content of 41.1%. A total of 10,910 protein-coding genes were predicted to be present in this genome using the Maker [1], and the N50 contig length was determined to be 1,983,291 bp. The data have been deposited in GenBank and can be viewed at https://www.ncbi.nlm.nih.gov/assembly/GCA_002196765.1.

**Table 1:** Genomic features of *M. farinosa* strains.

Attributes	KCTC27753	CBS7064
Total length (bp)	21,255,474	21,459,642
Contigs	17	18
N50	1,983,291	1,666,063
GC content (%)	41.1	41.4
Protein count	10,910	11,175
Reference	This study	[8]

The draft genome sequence of *M. farinosa* KCTC27753 provides a novel and useful source of genomic information regarding multi-stress-tolerant yeasts. This knowledge would be beneficial for applications in bioethanol production.

## 2 Experimental Design, Materials and Methods

Nuruk is a traditional Korean fermenting agent that contains numerous microorganisms, and its microbial community structure varies with its manufacturing conditions, such as the raw materials used and its manufacturing location [4]. To isolate yeast from nuruk, the fermenting agent it was homogenized with distilled water and then spread on yeast extract peptone dextrose (YEPD; yeast extract 10 g/L, peptone 20 g/L, glucose 20 g/L, BD, USA) medium containing chloramphenicol (100 mg/l, Sigma-Aldrich, Saint Lousis, MO, USA). *Millerozyma farinosa* KCTC27753 was isolated from the nuruk produced in Namhae, Gyeongsangbuk-do, South Korea. It was cultured in YEPD broth and incubated at 30 °C for 24 h. Cells were harvested with centrifugation(3000 rpm, 1 min) and then lysed using the cell lysis buffer and RNase A solution included in the G-DEX^TM^IIc Genomic DNA Extraction kit (iNtRON, Daejeon, Korea). Proteins in the cell lysate were extracted using the Protein PPT buffer included in the kit, and DNA was isolated using 100%(v/v) isopropanol (Sigma-Aldrich, USA) and 70%(v/v) ethanol (Duksan, Ansna, Korea).

The ITS region of the genomic DNA was amplified using ITS1 (5′-TCCGTAGGTGAACCTGCGG-3′) and ITS4 (5′-TCCTCCGCTTATTGATATGC-3′) primers and then sequenced. *Millerozyma farinosa* KCTC27753 was identified based on the results of the nucleotide sequence homology analysis performed using Basic Local Alignment Search Tool (BLAST, https://blast.ncbi.nlm.nih.gov/Blast.cgi) of NCBI. The ITS region of *M. farinosa* KCTC27753 genome was aligned with the ITS region from the genomes of other yeast strains in the GenBank database using the CLC Genomic Workbench (v20.0.4). The phylogenetic tree was constructed using the neighbor-joining method [9] with the MEGA (v.7.0.26).

The genome of *M. farinosa* KCTC27753 was sequenced using a PacBio RSII instrument(Pacific Biosciences, Menlo Park, CA, USA). The reads were assembled *de novo* using the FALCON (http://dazzlerblog.wordpress.com). Open reading frames (ORF) were predicted using the MAKER (http://www.yandell-lab.org/software/maker.html) and sub-annotated using the BLAST.

## Ethics Statements

Not applicable.

## CRediT authorship contribution statement

**Jeong-Ah Yoon:** Methodology, Software, Data curation, Writing – original draft, Visualization. **Yu-Jeong Lee:** Methodology, Software, Data curation, Writing – original draft, Visualization. **Eun-Hee Park:** Writing – review & editing, Data curation. **Hun-Joo Kwon:** Data curation. **Myoung-Dong Kim:** Conceptualization, Supervision, Funding acquisition.

## Declaration of Competing Interest

The authors declare that they have no known competing financial interests or personal relationships that could have appeared to influence the work reported in this paper.
